# Sonographic evaluation of children with congenital hypothyroidism*

**DOI:** 10.1590/0100-3984.2014.0040

**Published:** 2015

**Authors:** Anelise de Almeida Sedassari, Luis Ronan Marquez Ferreira de Souza, Nathalie de Almeida Sedassari, Maria de Fátima Borges, Heloisa Marcelina da Cunha Palhares, Genésio Borges de Andrade Neto

**Affiliations:** 1 Physician on duty at Hospital São Marcos de Uberaba, Uberaba, MG, Brazil.; 2 PhD, Associate Professor, Universidade Federal do Triângulo Mineiro (UFTM), Uberaba, MG, Brazil.; 3 MD, Resident in Medical Practice at Universidade Estadual Paulista “Júlio de Mesquita Filho” (Unesp), Botucatu, SP, Brazil.; 4 PhD, Associate Professor (Level IV), Service of Endocrinology, Universidade Federal do Triângulo Mineiro (UFTM), Uberaba, MG, Brazil.; 5 Master, MD, Service of Pediatric Endocrinology and Genetics, Universidade Federal do Triângulo Mineiro (UFTM), Uberaba, MG, Brazil.; 6 MD, Radiologist, Graduate Student degree of Ultrasonography, Computed Tomography and Magnetic Resonance Imaging, Hospital Israelita Albert Einstein, São Paulo, SP, Brazil.

**Keywords:** Congenital hypothyroidism, Ultrasonography, Main diagnosis

## Abstract

**Objective:**

To establish benchmarks and study some sonographic characteristics of the thyroid gland in a group of euthyroid children aged up to 5 years as compared with age-matched children with congenital hypothyroidism.

**Materials and Methods:**

Thirty-six children (17 female and 19 male) aged between 2 months and 5 years were divided into two groups – 23 euthyroid children and 13 children with congenital hypothyroidism – and were called to undergo ultrasonography.

**Results:**

In the group of euthyroid children (*n* = 23), mean total volume of the thyroid gland was 1.12 mL (minimum, 0.39 mL; maximum, 2.72 mL); a homogeneous gland was found in 17 children (73.91%) and 6 children (26.08%) had a heterogeneous gland. In the group of children with congenital hypothyroidism (*n* = 13), mean total volume of the thyroid gland was 2.73 mL (minimum, 0.20 mL; maximum, 11.00 mL). As regards thyroid location, 3 patients (23.07%) had ectopic thyroid, and 10 (69.23%) had topic thyroid, and out of the latter, 5 had a homogeneous gland (50%) and 5, a heterogeneous gland (50%). In the group with congenital hypothyroidism, 6 (46.15%) children had etiological diagnosis of dyshormoniogenesis, 3 (23.07%), of ectopic thyroid, and 4 (30.76%), of thyroid hypoplasia.

**Conclusion:**

Thyroid ultrasonography is a noninvasive imaging method, widely available, easy to perform and for these reasons could, and should, be performed at any time, including at birth, with no preparation or treatment discontinuation, to aid in the early etiological definition of congenital hypothyroidism.

## Abstract (pt)

**Objetivo:**

Estabelecer padrões de referência e estudar algumas características ultrassonográficas tireoidianas em um grupo de crianças eutireoidianas até os 5 anos de idade, comparando-as a crianças portadoras de hipotireoidismo congênito de mesma faixa etária.

**Objetivo:**

Trinta e seis crianças foram divididas em dois grupos – 23 eutireoidianas e 13 portadoras de hipotireoidismo congênito – e convocadas para a realização de ultrassonografia de tireoide. Dessas crianças, 17 eram do sexo feminino e 19, do sexo masculino, com idades entre 2 meses e 5 anos.

**Resultados:**

No grupo de crianças eutireoidianas (*n* = 23), o volume total médio da glândula tireoide foi 1,12 mL (mínimo: 0,39 mL; máximo: 2,72 mL), sendo observadas, também, glândula homogênea em 17 crianças (73,91%) e glândula heterogênea em 6 crianças (26,08%). No grupo de crianças com hipotireoidismo congênito (*n* = 13), o volume total médio da glândula tireoide foi 2,73 mL (mínimo: 0,20 mL; máximo: 11,00 mL). Quanto à localização da tireoide, foram encontradas 3 crianças (23,07%) com tireoide ectópica e 10 (69,23%) com tireoide tópica, sendo que, nessas últimas, 5 tinham glândula homogênea (50%) e 5, glândula heterogênea (50%). Das crianças com hipotireoidismo congênito, 6 (46,15%) apresentavam diagnóstico etiológico de disormoniogênese, 3 (23,07%) tinham diagnóstico etiológico de ectopia e 4 (30,76%) possuíam diagnóstico etiológico de hipoplasia tireoidiana.

**Conclusão:**

A ultrassonografia de tireoide, por se tratar de um método não invasivo, bastante disponível, de fácil realização, pode e deve ser realizada a qualquer momento, inclusive ao nascimento, sem preparação ou interrupção do tratamento, para auxiliar na definição etiológica precoce do hipotireoidismo congênito.

## INTRODUCTION

In the early days of life, thyroid ultrasonography (TUS) may be performed in the context of congenital hypothyroidism, assisting in the etiological diagnosis. Thyroid dysgenesis includes athyreosis, as an “empty” thyroid area either with ectopic tissue or not (agenesis), and thyroid hypoplasia. Cases where the thyroid gland is topic at US, with normal or increased volume, suggest one of the various presentations of dyshormonogenesis^(1)^.

A child with a confirmed congenital hypothyroidism diagnosis requires immediate treatment with L-thyroxine, and the etiological investigation is delayed until the age of three years. Many parents become apprehensive because of the lack of a definition on the cause of congenital hypothyroidism and with such a delay. The use of thyroid scintigraphy, considered as being the gold standard for localization of the gland, is not recommended in the neonatal period, and when it is later performed, medication discontinuation is required. Additionally, the gland volume will be reduced due to the utilization of L-thyroxine, possibly leading to a false diagnosis of hypoplasia^(2-4)^.

The etiological diagnosis may be deepened by means of a molecular study of the congenital thyroid defect, and TUS can guide the early evaluation, without the need for therapy interruption. According to the thyroid location obtained at TUS, the patients may be referred to molecular investigation of thyroid dysgenesis or dyshormonogenesis without the need for scintigraphy and, consequently, therapy interruption, with more comfort to the affected individuals. TUS is an excellent method to localize the thyroid gland, with the advantage that it can be utilized in the neonatal period. However, there are no reference standards for the interpretation of such an imaging study at early age ranges^(5,6)^.

The present study is aimed at establishing reference standards as well as studying some sonographic thyroid features in a group of euthyroid children up to five years of age, comparing them with children presenting with congenital hypothyroidism in the same age group.

## MATERIALS AND METHODS

The present study was duly approved by the Committee for Ethics in Research of Universidade Federal do Triângulo Mineiro (UFTM). The children’s parents or caretakers signed a Term of Free and Informed Consent, authorizing the children to be submitted to TUS.

Euthyroid children were selected at the Childcare Clinic – Pediatrics Division of UFTM. The children who were found to be healthy at examination and with normal neonatal heel prick (Guthrie test) were included in the study. This study sample comprised 23 children (12 girls and 11 boys), with ages ranging from 2 months to 5 years, distributed into groups of 5 children each in the following age ranges – 1 year and 1 month to 2 years; 3 years and 1 month to 4 years; and 4 years and 1 month to 5 years –, and groups of 4 children each in the following age ranges – 2 months to 1 year and 2 years; and 1 month to 3 years.

The children presenting with congenital hypothyroidism were selected at the ambulatory of the Endocrinology Division of UFTM. The selected children were those with positive Guthrie test and subsequent confirmation by means of hormone testing, and all of them undergoing treatment with individualized doses of thyroid hormone.

Thirteen out of a total of 32 children under current and regular follow-up at the service of endocrinology were selected, 5 of them being girls and 8 being boys, with ages ranging between 2 months and 5 years (3 children between 2 months and 1 year; 2 children between 1 year and 1 month to 2 years; 3 children between 2 years and 1 month to 3 years; 1 child between 3 years and one month to 4 years; and 4 children between 4 years and 1 month to 5 years). The study included 17 female children (47.22%) and 19 male children (52.77%).

**Figure 1:**
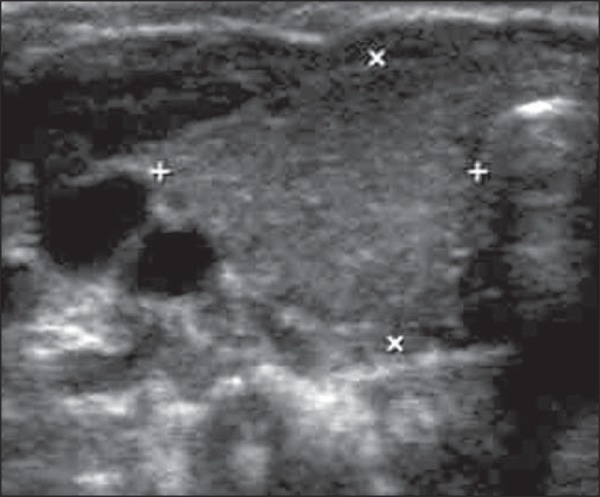
Ultrasonography of the cervical region in an asymptomatic child. Observe the right thyroid lobe (between markers) with preserved echotexture and dimensions.

All the children participating in the study underwent TUS for evaluation of the gland´s characteristics such as position, texture, volume and additional findings (Figure 1). The scans were carried out with the patients in dorsal decubitus and neck hyperextension in order to facilitate the anatomical analysis.

The scans started with the evaluation of the thyroid, characterizing its lobes and the isthmus, location in the neck, echotexture, dimensions and volume. The thyroid volume was calculated by the apparatus itself by means of the builtin standard formula in the preset mode for thyroid, with measurements on the longitudinal section (craniocaudal and anteroposterior) and on cross-sectional section for the remaining measurements.

In some cases, the behavior at color Doppler was also analyzed. In cases where a thyroid lesion was identified, it was recorded and separately analyzed.

The US scans were performed at the Unit of Radiology and Imaging Diagnosis of Hospital de Clínicas – UFTM, with a HD11 US apparatus (Philips Medical Systems; Bothell, WA, USA), equipped with a 7.5 MHz (3–13 MHz) linear transducer, and an Accuvix V10 model (Samsung Medison America; Cypress, CA, USA), equipped with a linear 7.5 MHz (6–12 MHz) transducer.

### Statistical analysis

The thyroid volumes were recorded as mean ± standard deviation and also as minimum and maximum values. The Student’s t test was utilized in the comparison of the thyroid volumes between the two groups, euthyroid children and those presenting with congenital hypothyroidism at each age group. Values corresponding to *p* < 0.05 were considered to be significant.

## RESULTS

In the group of euthyroid children (*n* = 23), the mean total volume of the thyroid gland was 1.12 mL (minimum: mL; maximum: 2.72 mL), with the following distribution according age groups: 0.62 mL (0.52–0.70) in 4 children between 2 months and 1 year of age; 0.77 mL (0.39– 1.29) in 5 children between 1 year and one month to 2 years of age; 0.78 mL (0.65–0.88) in 4 children between 2 years and 1 month to 3 years of age; 1.30 mL (0.98–1.70) in 5 children between 3 years and 1 month to 4 years of age; and mL (1.39–2.72) in 5 children between 4 years and 1 month to 5 years. Additionally, homogeneous thyroid was observed in 17 children (73.91%) while in 6 children (26.08%) the thyroid was heterogeneous.

In the group of children with congenital hypothyroidism (*n* = 13), the mean total thyroid volume was 2.73 mL (0.20–11.00) distributed according age groups, as follows: 0.82 mL (0.20–3.00) in 3 children between 2 months and 1 year of age; 1.30 mL (1.90–2.20) in 2 children between 1 year and one month and 2 years of age; 7.75 mL (4.50–11.00) in 3 children between 2 years and one month and 3 years of age; 2.5 mL in one child between 3 years and 1 month and 4 years of age; and 0.67 mL (0.20-1.00) in 4 children between 4 years and 1 month and 5 years of age.

As regards *p* values (Student’s *t* test), comparing euthyroid children with those presenting with congenital hypothyroidism, the mean *p* value was 0.001, distributed according age ranges as follows: 0.384 in 7 children between 2 months and 1 year of age; 0.051 in 7 children between 1 year and 1 month to 2 years of age; 0.008 in 7 children between 2 years and one month to 3 years of age; 0.007 in 9 children between 4 years and one month to 5 years of age. It was not possible to perform the calculation for 6 children between 3 years and one month to 4 years of age.

**Figure 2:**
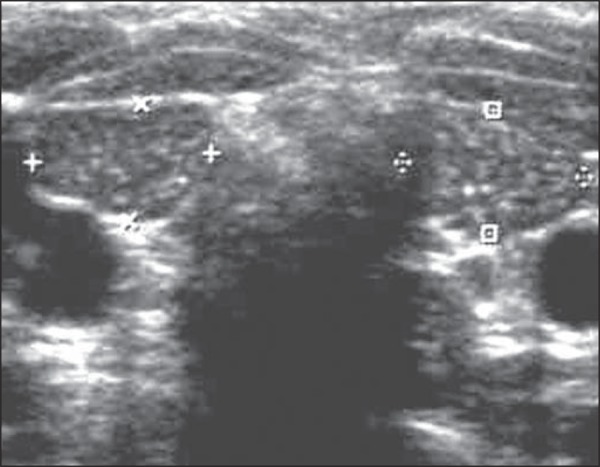
Ultrasonography of cervical region. On this image, the thyroid is hyperechogenic and presents with reduced dimensions (between markers).

**Figure 3:**
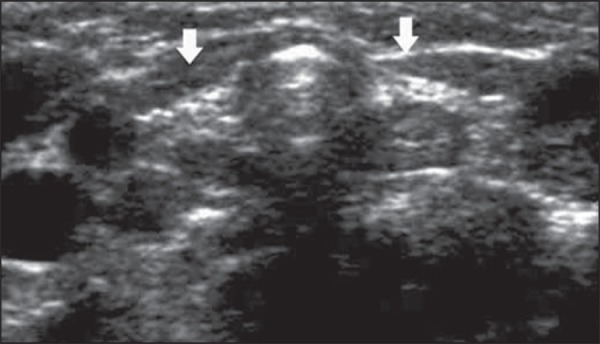
Ultrasonography of cervical region. In this case, the thyroid gland (arrows indicating the right and left lobes) presents with much reduced dimensions and increased echogenicity.

As regards location of the thyroid, 3 patients (23.07%) presented with ectopic thyroids while 10 patients (76.92%) presented with topic thyroids, among them 5 patients had homogeneous glands (50%) and 5, heterogeneous glands (50%) (Figures 2 and 3).

**Figure 4:**
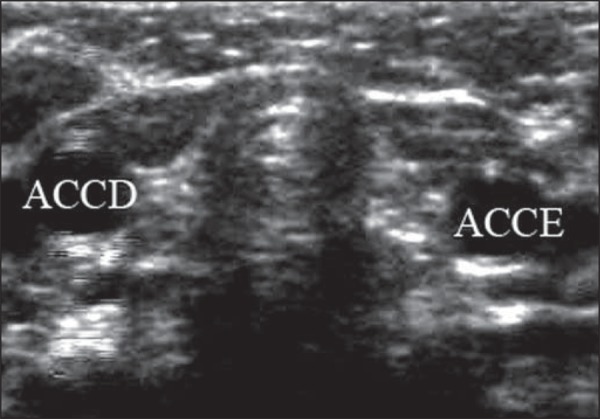
Ultrasonography of cervical region. In this child, the thyroid gland could not be identified. ACCD, right common carotid artery; ACCE, left common carotid artery.

**Figure 5:**
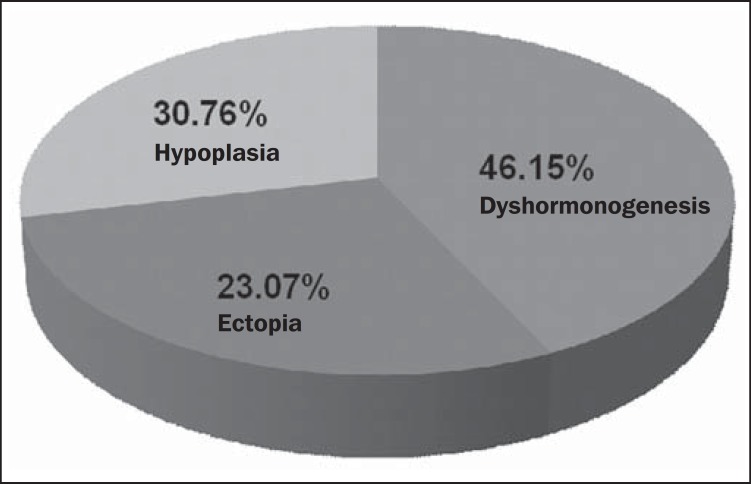
Etiological diagnosis of congenital hypothyroidism.

As regards the data from TUS scans and follow-up performed in the ambulatory, the authors observed that 6 children (46.15%) presented with etiological diagnosis of dyshormonogenesis, 3 children (23.07%) presented with etiological diagnosis of ectopia and 4 children (30.76%) presented with etiological diagnosis of thyroid hypoplasia (Figures 4 and 5).

## DISCUSSION

The neonatal screening programs for congenital hypothyroidism by means of the Guthrie test have demonstrated a relevant prevalence of congenital hypothyroidism among the different populations, and some studies suggest ethnic variation, with higher prevalence among the Hispanic and Native American populations (1:2,000) and lower prevalence among black individuals (1:10,000). Recent local studies indicated a prevalence of 1: 2,017, with dyshormonogenesis as the main cause, demonstrating both increased prevalence and a shift in etiological pattern in the region^(7)^.

Such figures, associated with the knowledge that thyroid hormone is essential for the neonatal neurological development, justify efforts in the search of simple and effective methods to aid in the etiological definition of congenital hypothyroidism^(8)^. TUS, for being a non-invasive, widely available and easily performed method, could be performed at any moment including at birth, with no preparation or treatment interruption to aid in the early etiological definition of congenital hypothyroidism^(9,10)^.

However, there are few studies in the literature regarding reference values for volume and thyroid features in healthy children, moreover in age groups below the age of 5, the period where the thyroid hormone administration is interrupted in order to perform the tests for etiological definition, such as scintigraphy, perchlorate test and others. The present study included younger children, aged between 2 months and 1 year, considering that TUS can be the first scan to be performed before the administered thyroid hormones reduce the thyroid volume to the point of suggesting a false diagnosis of thyroid hypoplasia^(8,11,12)^.

In the group of 23 euthyroid children, the mean total volume of the gland was 1.12 ± 0.56 mL, but increasing and significant thyroid volumes were observed as the children advanced one year in the age range, justifying the stratification by age adopted in the present study. In the comparison between the euthyroid children and the congenital hypothyroidism group, the authors also observed that the volumes did not differ in the first year of life, but were significantly greater in the children presenting with congenital hypothyroidism and topic thyroid.

Most euthyroid children presented with homogeneous thyroid (73.91%), while 50% of the children with congenital hypothyroidism demonstrated images heterogeneity. There was agreement with the previous diagnosis of ectopia obtained by means of scintigraphy in three children, while four of the children presented with thyroid that were small for their age range, indicating hypoplasia; however as they were being administered thyroid hormones since birth, it is very likely that the small size of the gland was an effect of the medication. In a previous study, the authors demonstrated, by means of TUS performed at different intervals, that thyroid medication can reduce thyroid volumes in children presenting with congenital hypothyroidism.

Therefore, the diagnosis of thyroid hypoplasia would be more reliable if made at birth or within the first year of life.

In the literature, some studies point towards thyroid dysgenesis, mainly ectopia^(9)^, as the main cause for congenital hypothyroidism, while others^(10)^, including the present study, have found a higher prevalence of dyshormonogenesis. It is very likely that local and environmental factors yet to be defined are responsible for such discrepancies. In any of such situations, TUS has demonstrated to be an excellent tool to localize the thyroid gland, with the advantage that scans can be repeatedly performed in the neonatal period^(13)^.

The deepening of the etiological diagnoses will come with the molecular study of the congenital thyroid defect. Not so long ago, thyroid dysgenesis itself was considered to be a sporadic event. Over the last years, reports of familial cases with multiple affected members, and studies on molecular biology have demonstrated the involvement of genes (TTF1 and 2, PAX8, TSH-R) which codify highly conserved transcription factors which, if inactivated, result in thyroid agenesis, ectopia and hypoplasia. Additionally, the description of other affected genes responsible for the cases of dyshormonogenesis and transient hyperthyreotropinemia will make etiological diagnosis much more practical and objective. According to the thyroid location obtained at TUS, the patients can be referred to molecular investigation of thyroid dysgenesis or dyshormonogenesis without the need for scintigraphy and therapy interruption, with much more comfort for the affected individuals^(13-15)^.

The authors conclude that TUS can and should be performed right after birth, as soon as the Guthrie test indicates a situation of congenital hypothyroidism and the treatment is instituted, since, based the presence of topic or ectopic thyroid, it can define and suggest the cause of congenital hypothyroidism. Also, the reference services should have their normality values classified by age group. For such a reason, it is important that further studies be undertaken, approaching reference values for thyroid volume and characteristics in healthy children (particularly in age ranges under 5 years of age) in order to define a standard for such characteristics. In the future, sonographic findings may be associated with the molecular diagnosis to aid in an earlier genetic counseling than it is currently possible.
